# Exploration of the Mechanism of Linoleic Acid Metabolism Dysregulation in Metabolic Syndrome

**DOI:** 10.1155/2022/6793346

**Published:** 2022-11-28

**Authors:** Yan Wen, Yawen Shang, Qing Wang

**Affiliations:** Department of Endocrinology, China-Japan Union Hospital of Jilin University, 126 Xian-tai Street, Changchun, Jilin 130033, China

## Abstract

We aimed to explore the mechanism of the linoleic acid metabolism in metabolic syndrome (MetS). RNA-seq data for 16 samples with or without MetS from the GSE145412 dataset were collected. Gene set variation analysis (GSVA), gene set enrichment analysis (GSEA), and gene differential expression analysis were performed. Expression data of differentially expressed genes (DEGs) involved in the linoleic acid metabolism pathway were mapped to the pathway by using Pathview for visualization. There were 19 and 10 differentially expressed biological processes in the disease group and healthy group, respectively. 9 KEGG pathways were differentially expressed in the disease group. Linoleic acid metabolism was the only differentially expressed pathway in the healthy group. The GSVA enrichment score of the linoleic acid metabolism pathway in the disease group was markedly lower than that in the healthy group. The GSEA result showed that the linoleic acid metabolism pathway was significantly downregulated in the disease group. JMJD7-PLA2G4B, PLA2G1B, PLA2G2D, CYP2C8, and CYP2J2 involved in the pathway were significantly downregulated in the disease group. This study may provide novel insight into MetS from the point of linoleic acid metabolism dysregulation.

## 1. Introduction

Metabolic syndrome (MetS) is a common complex entity characterized by a set of metabolic abnormalities that serve as risk factors for the development of cardiovascular disease (CVD) and type 2 diabetes mellitus (T2DM) [1]. MetS doubles the risk of CVD and increases the risk of diabetes five-fold [2, 3]. The main characteristic components of MetS include central obesity, hypertension, hyperglycemia, hypertriglyceridemia, and a low level of high-density lipoprotein cholesterol (HDL-C) [1]. The existence of any three or more of these traits constitutes a clinical diagnosis of MetS. The prevalence of MetS ranges from 10% to 40% and keeps rising with the increasing rate of obesity worldwide [4]. The complicated pathogenesis of MetS results from the interaction of multiple biological processes (BPs) and pathways, as well as genetic variations and environmental and nutritional factors; however, the exact underlying mechanisms have not been fully understood [5].

As an essential nutrient, linoleic acid is the most abundant n-6 polyunsaturated fatty acid (PUFA) in the human diet [6]. PUFAs are the basic components of cellular membranes and serve as cellular signaling molecules. Linoleic acid usually plays an active role in human health. Linoleic acid is correlated with a reduced incidence of CVD and T2DM, which may be attributed to its action on risk factors for diseases, for instance, linoleic acid's reduction of blood cholesterol levels and its impact on insulin and glucose metabolisms [7]. Therefore, linoleic acid may be associated with MetS.

Gene set enrichment methods focus on gene sets in the analysis of gene expression data. Gene set variation analysis (GSVA) calculates gene set enrichment scores for each sample and assesses the variation of gene set enrichment over the samples. It identifies the differential pathway activity in an unsupervised manner [8]. Gene set enrichment analysis (GSEA) utilizes the predefined gene sets and ranks of genes to determine the significant pathways that are related to the phenotypic distinction [9]. Compared with single-gene analysis, gene set enrichment methods have several benefits, including dimensionality reduction and interpretability [10]. In the present study, we performed GSVA, GSEA, and gene differential expression analysis on RNA-seq data of 16 samples from the GSE145412 expression profile [11]. 16 samples were divided into four groups according to their metabolic health status and body mass index (BMI), including lean (BMI < 25) with the MetS group (MetS lean) and obese (BMI > 30) with the MetS group (MetS obese), the healthy obese group, and the healthy lean group [11]. We found that the enrichment score of the linoleic acid metabolism pathway in the MetS samples was markedly lower than that in the healthy controls, suggesting the dysregulation of the linoleic acid metabolism pathway in MetS, and JMJD7-PLA2G4B, PLA2G1B, PLA2G2D, CYP2C8, and CYP2J2 involved in the pathway were significantly downregulated. This study may provide novel insight into MetS from the point of linoleic acid metabolism dysregulation.

## 2. Materials and Methods

### 2.1. Data Collection and Preprocessing

Raw data from the GSE145412 dataset [11] were downloaded from the Sequence Read Archive (SRA). FASTX-Toolkit was used to trim off the low-quality bases of the raw reads. After quality control by FastQC, clean reads were mapped to the reference genome via TopHat2 (*Homo sapiens*, GRCh38, version 23). Single-mapped reads were utilized to calculate the read count and TPM for each gene. TPM normalization was performed by the normalizeBetweenArrays function of the limma package [12].

### 2.2. GSVA and GSEA

Gene sets in *Homo sapiens* grouped by KEGG pathways and gene ontology BPs were downloaded from the Molecular Signatures Database (MSigDB) [13] by using the msigdbr R package. The enrichment scores of each sample on gene sets were computed by the GSVA R package. Differentially expressed gene sets were identified by the limma package. The thresholds of differentially expressed BPs were set as |log_2_FC| > 0.5 and *p* < 0.05, while those of differentially expressed pathways were set as |log_2_FC| > 0.1 and *p* < 0.05. Significantly enriched pathways were identified by the GSEA method with the cutoff values of NOM *p* value <0.05 and FDR *q* value <0.25.

### 2.3. Identification of Differentially Expressed Genes (DEGs)

The gene set related to the linoleic acid metabolism pathway was extracted from MSigDB to construct a protein-protein interaction (PPI) network by using STRING, in which genes with the top 10 highest degrees were regarded as hub genes [14]. DEGs between groups were identified by using the Wilcoxon rank sum test with the threshold as *p* < 0.05. Expression data of DEGs were mapped to the linoleic acid metabolism pathway for visualization by using Pathview [15], and proteins encoded by DEGs were colored in the graph to display their roles in the linoleic acid metabolism pathway.

### 2.4. Statistical Analysis

All the statistical analyses were performed by R Statistical Software (version 4.1.2). The Wilcoxon rank sum test was applied to compare the differences between groups. *p* < 0.05 was statistically significant.

## 3. Results

### 3.1. Significantly Downregulated Linoleic Acid Metabolism Pathway in MetS

**Figure 1:**
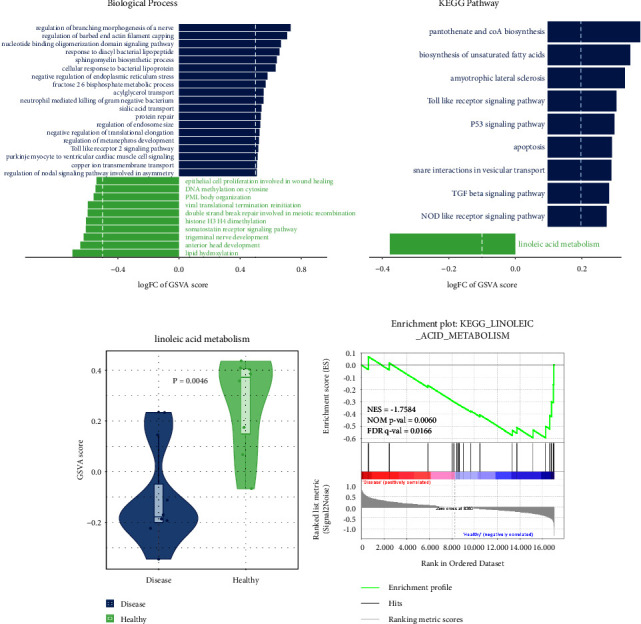
Gene set enrichment analysis. (a) Differentially expressed BPs in the disease group and the healthy group. (b) Differentially expressed pathways in the disease group and the healthy group. (c) Comparison of the GSVA enrichment score of the linoleic acid metabolism pathway between the disease group and the healthy group. (d) GSEA result of the linoleic acid metabolism pathway in the disease group and the healthy group.

16 samples were divided into four groups, including MetS lean, MetS obese, healthy obese, and healthy lean. Differentially expressed gene sets are shown in Figure S1. There were 19 differentially expressed BPs in the disease group, including regulation of nodal signaling pathways involved in asymmetry, copper ion transmembrane transport, Purkinje myocyte to ventricular cardiac muscle cell signaling, Toll-like receptor 2 signaling pathway, regulation of metanephros development, negative regulation of translational elongation, regulation of endosome size, protein repair, sialic acid transport, the neutrophil-mediated killing of gram-negative bacteria, acylglycerol transport, fructose 2,6-bisphosphate metabolic process, negative regulation of endoplasmic reticulum stress, cellular response to bacterial lipoprotein, sphingomyelin biosynthetic process, response to diacyl bacterial lipopeptide, nucleotide-binding oligomerization domain signaling pathway, regulation of barbed end actin filament capping, and regulation of branching morphogenesis of a nerve (Figure 1(a)). In the healthy group, there were 10 differentially expressed BPs, including lipid hydroxylation, anterior head development, trigeminal nerve development, the somatostatin receptor signaling pathway, histone H3 H4 dimethylation, double-strand break repair involved in meiotic recombination, viral translational termination reinitiation, PML body organization, DNA methylation on cytosine, and epithelial cell proliferation involved in wound healing (Figure 1(a)). On the other hand, differentially expressed pathways in the disease group involved pantothenate and coA biosynthesis, biosynthesis of unsaturated fatty acids, amyotrophic lateral sclerosis, the Toll-like receptor signaling pathway, the P53 signaling pathway, apoptosis, snare interactions in vesicular transport, the TGF beta signaling pathway, and the NOD-like receptor signaling pathway, while in the healthy group, linoleic acid metabolism was the only differentially expressed pathway (Figure 1(b)). The GSVA enrichment score of the linoleic acid metabolism pathway was markedly lower in the disease group than that in the healthy group (Figure 1(c)). The GSEA result revealed that the linoleic acid metabolism pathway was significantly downregulated in the disease group (Figure 1(d)).

**Figure 2:**
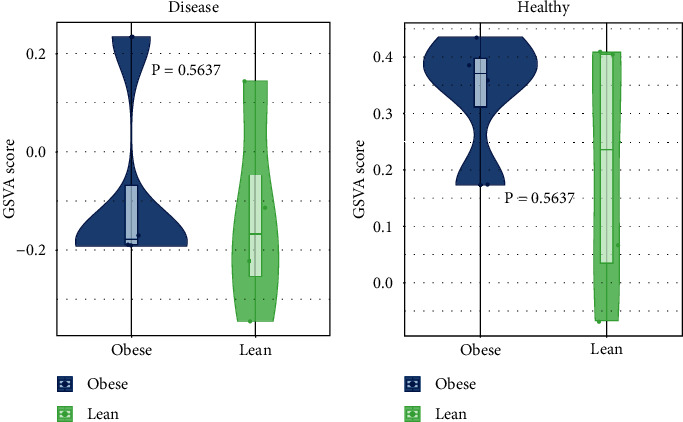
Comparison of the GSVA enrichment score of the linoleic acid metabolism pathway between obese and lean samples in the disease group (a) and the healthy group (b).

We next examined the differences in the GSVA enrichment score of the linoleic acid metabolism pathway between obese and lean samples in the disease group and the healthy group. As depicted in Figure 2, there was no significant difference in the GSVA enrichment score of the linoleic acid metabolism pathway between obese and lean samples in both groups, suggesting that linoleic acid metabolism dysregulation was not significantly associated with obesity.

**Figure 3:**
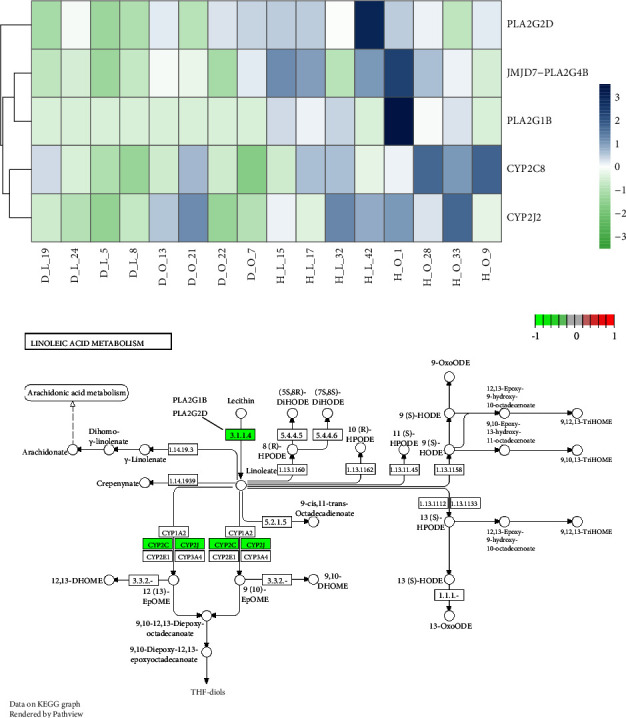
Linoleic acid metabolism pathway visualization. (a) Significantly downregulated genes involved in the linoleic acid metabolism pathway. (b) Linoleic acid metabolism pathway visualization with mapped data of DEGs.

29 genes involved in the linoleic acid metabolism pathway were obtained, and the PPI network is shown in Figure S2A. The hub genes with the top 10 highest degrees were ALOX15, CYP2J2, CYP2C8, CYP3A4, CYP2C9, CYP1A2, CYP2C19, PLA2G3, PLA2G12A, and CYP2E1 (Figure S2B). Several genes were found to be significantly downregulated in the linoleic acid metabolism pathway, including JMJD7-PLA2G4B, PLA2G1B, PLA2G2D, CYP2C8, and CYP2J2 (Figure 3(a)), and DEG-encoded proteins participated in the linoleic acid metabolism pathway as enzymes (Figure 3(b)).

## 4. Discussion

MetS has become a global health concern currently. Except for heart disease and diabetes, MetS also has a link with cancer [16]. The complex interplay of multiple causative factors makes it hard to treat MetS [17]. A healthy lifestyle (including diet and physical activity), weight loss, and control of comorbidities are the therapeutic targets of MetS, and long-term goals include reducing the risks of CVD, T2DM, and other MetS-related conditions [18]. The enhancement of knowledge on mechanisms of MetS contributes to improving interventions and treatments for MetS. Fatty acids are a major source of energy. As mediators for cell signaling, they play a role in the etiology of MetS [19].

In the present study, we applied GSVA and GSEA to the RNA-seq data from patients with or without MetS and found that the enrichment score of the linoleic acid metabolism pathway was significantly lower in MetS patients, suggesting the dysregulation of the linoleic acid metabolism pathway in MetS. Through integrated proteomics and metabolomics analysis, Chen et al. also revealed that the linoleic acid metabolism pathway was significantly perturbed in the MetS rat model [20]. Szczuko et al. observed a significant decrease in the content of linoleic acid in MetS compared with the healthy control [21]. Linoleic acid has a correlation with metabolic diseases [22]. Previous studies have revealed that lower linoleic acid content is associated with an increased risk for CVD and T2DM [23, 24]. Yary et al. found that higher linoleic acid concentrations were associated with a lower risk of developing MetS [25].

We also found that several genes involved in the linoleic acid metabolism pathway were significantly downregulated in MetS patients, including JMJD7-PLA2G4B, PLA2G1B, PLA2G2D, CYP2C8, and CYP2J2. PLA2G4B, PLA2G1B, and PLA2G2D encode members of the phospholipase A2 family that generally hydrolyze the sn-2 acyl bond in phospholipids to release fatty acids and lysophospholipids. In the linoleic acid metabolism pathway, lecithin in the cell membrane is converted by phospholipase A2 enzymes to linoleate. PLA2G4B was associated with age-related changes in phospholipids and decreased energy metabolism in monocytes [26]. PLA2G2D increased energy expenditure and thermogenesis by promoting adipocyte browning, thereby improving diet-induced metabolic disorders [27]. PLA2G1B deficiency or inactivation gave protection from diet-induced obesity, insulin resistance, hyperglycemia, hyperlipidemia, and atherosclerosis [28–30], and PLA2G1B inhibitors suppressed diet-induced obesity and diabetes effectively in mice [31], implying that PLA2G1B inhibition may be a viable therapeutic option for metabolic diseases.

CYP2C8 and CYP2J2 encode members of the cytochrome P450 (CYP) superfamily of enzymes. CYP monooxygenases participate in the metabolism of various endogenous substrates, including PUFAs. In a CYP-dependent manner, linoleic acid is metabolized to produce 9,10-epoxyoctadecenoic acid (9,10-EpOME) and 12,13-epoxyoctadecenoic acid (12,13-EpOME) [32]. CYP2C8 and CYP2J2 are two of the main CYP isoforms that catalyze this conversion. Subsequently, these epoxides are hydrolyzed by soluble epoxide hydrolase (sEH) to yield 9,10-dihydroxyoctadecenoic acid (9,10-DiHOME) and 12,13-dihydroxyoctadecenoic acid (12,13-DiHOME) [32]. These metabolites of CYP produced from linoleic acid are associated with health and disease in many ways [33]. 9,10-EpOME and 9,10-DiHOME induce oxidative stress under 90 *μ*M concentration by activating NF-*κ*B and AP-1 transcription factors that mediate inflammation [34]. EpOMEs and DiHOMEs exhibited cardiotoxicity [35, 36], while sEH inhibitors showed a cardioprotective effect [37–39]. In addition, the application of sEH inhibitors in MetS treatment has been explored. In a diet-induced MetS rat model, oral treatment with an sEH inhibitor alleviated the symptoms of MetS, indicating that sEH inhibitors have therapeutic potential for MetS [40].

The study has several limitations. First, the sample size was small. Second, the bioinformatic analysis was restricted to the RNA-seq data of MetS, while proteomics and metabonomics may provide novel insight into the underlying mechanisms of MetS. Third, there is a lack of experiments to detect the linoleic acid metabolism pathway's activity. Therefore, future studies should be conducted with a larger sample size and in a more comprehensive way, combining multidimensional omics data analysis and experimental verification to enhance the understanding of MetS.

## 5. Conclusion

In conclusion, we found the dysregulation of the linoleic acid metabolism pathway in MetS, and JMJD7-PLA2G4B, PLA2G1B, PLA2G2D, CYP2C8, and CYP2J2 involved in the linoleic acid metabolism pathway were significantly downregulated.

## Data Availability

The data used and/or analyzed during the current study are available from the corresponding author upon request.

## Supplementary Materials

Supplementary Materials Supplementary figures depict the differentially expressed gene sets (Figure S1) and the PPI network (Figure S2).
