# Supplementary figures and images for: Exploration of the Mechanism of Linoleic Acid Metabolism Dysregulation in Metabolic Syndrome
Source: Genet Res (Camb). 2022 Nov 28;2022:6793346. doi: 10.1155/2022/6793346 (PMC9722286; doi:10.1155/2022/6793346)

A

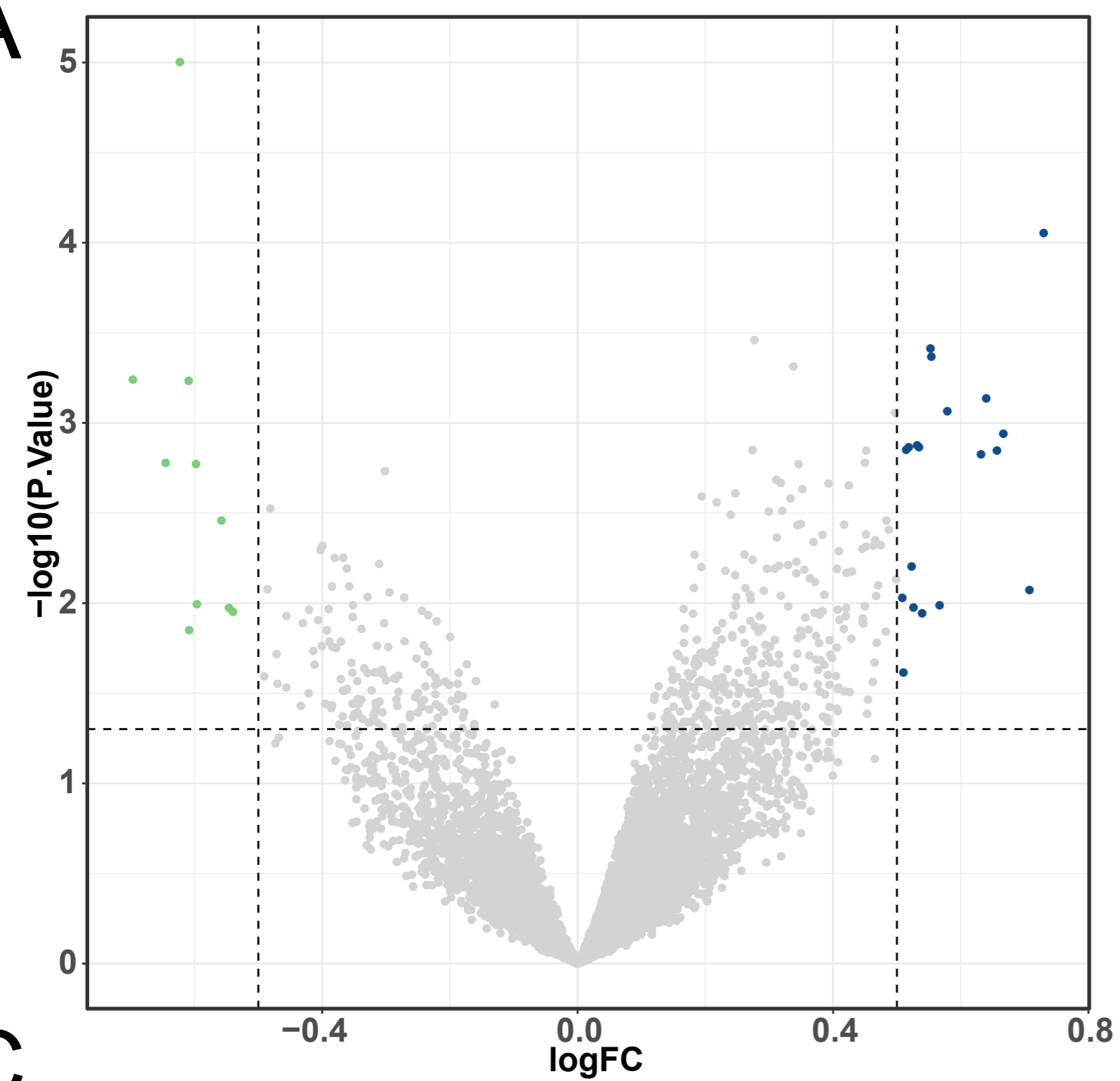

B

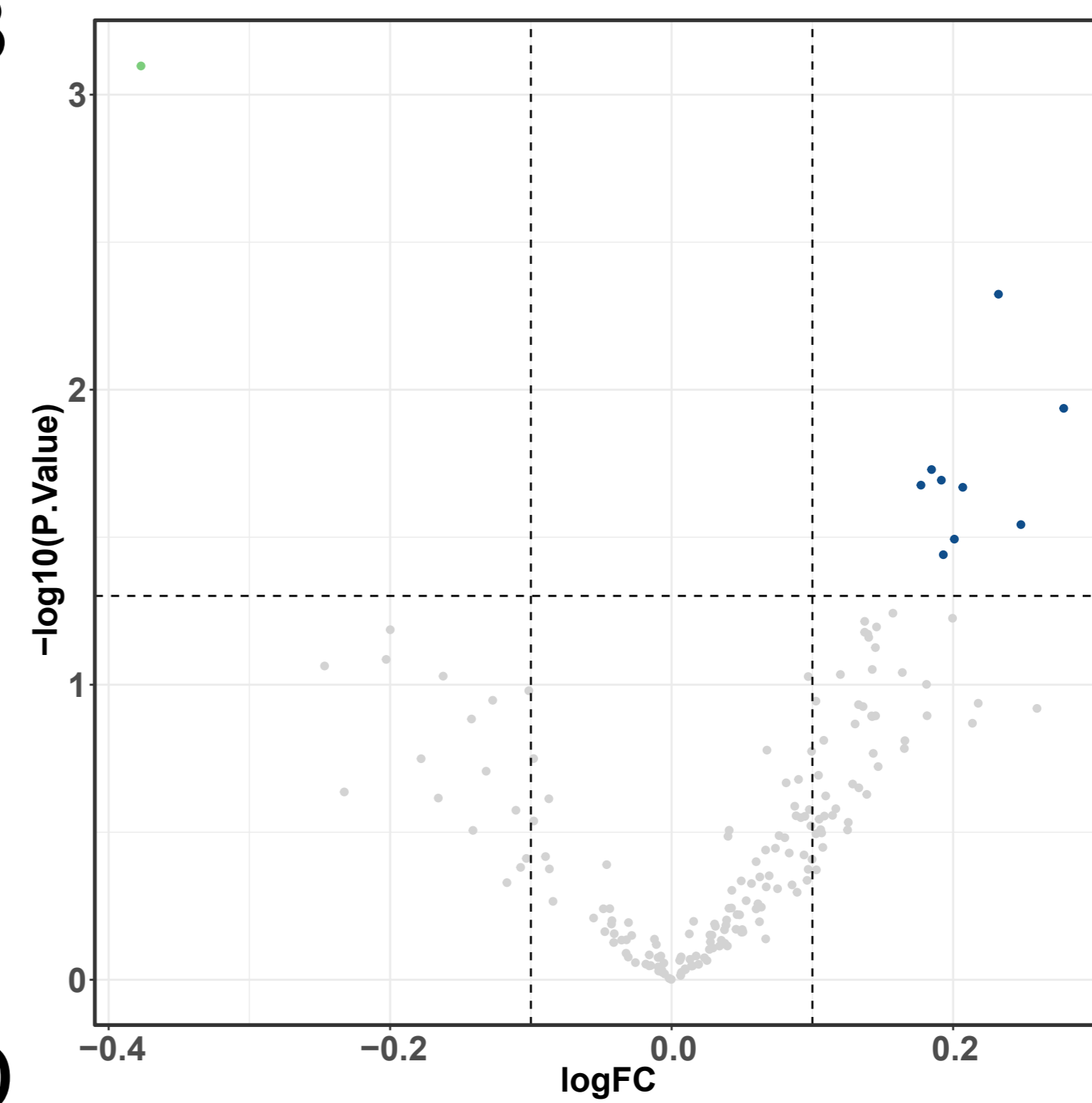

C

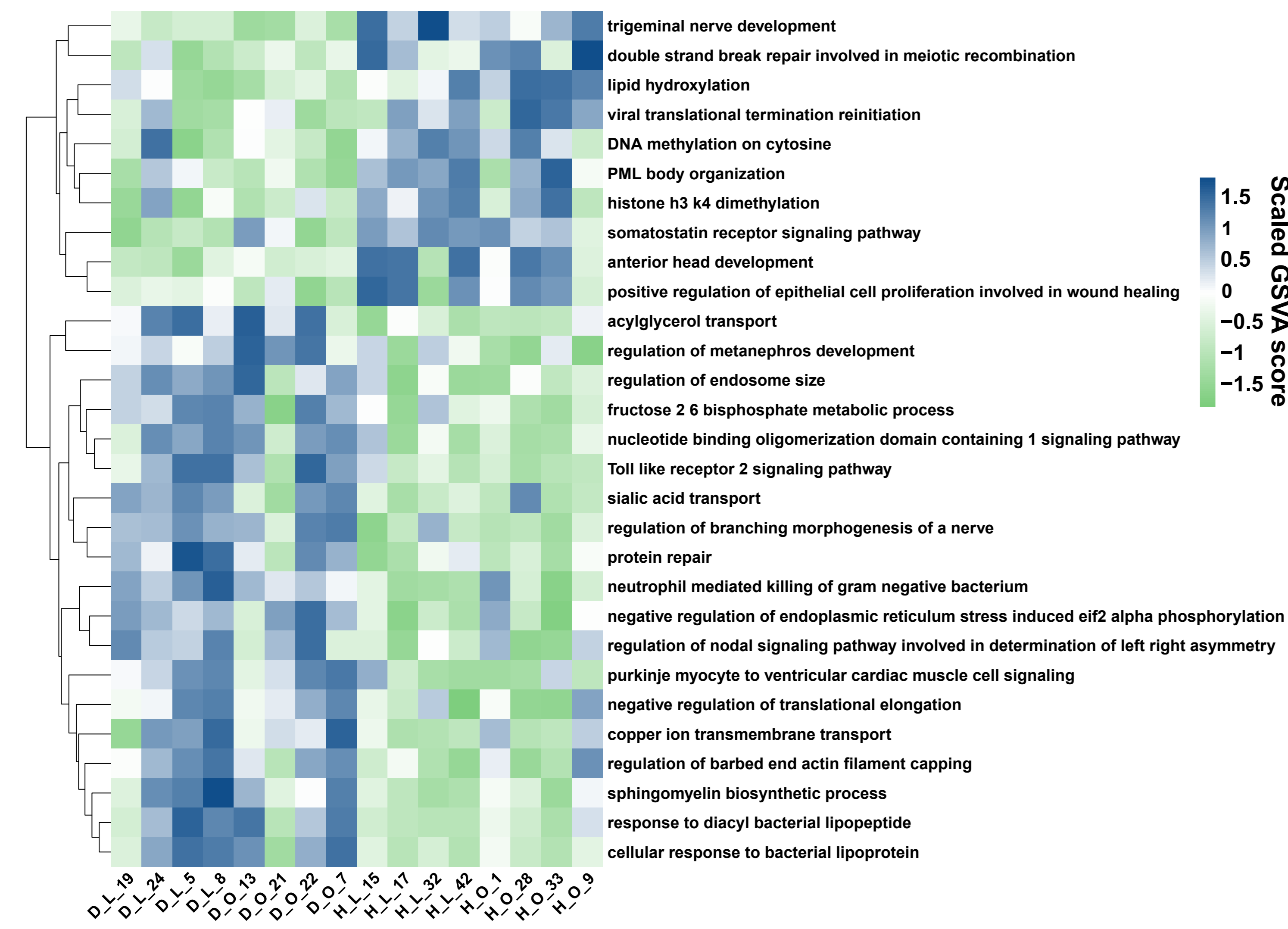

D

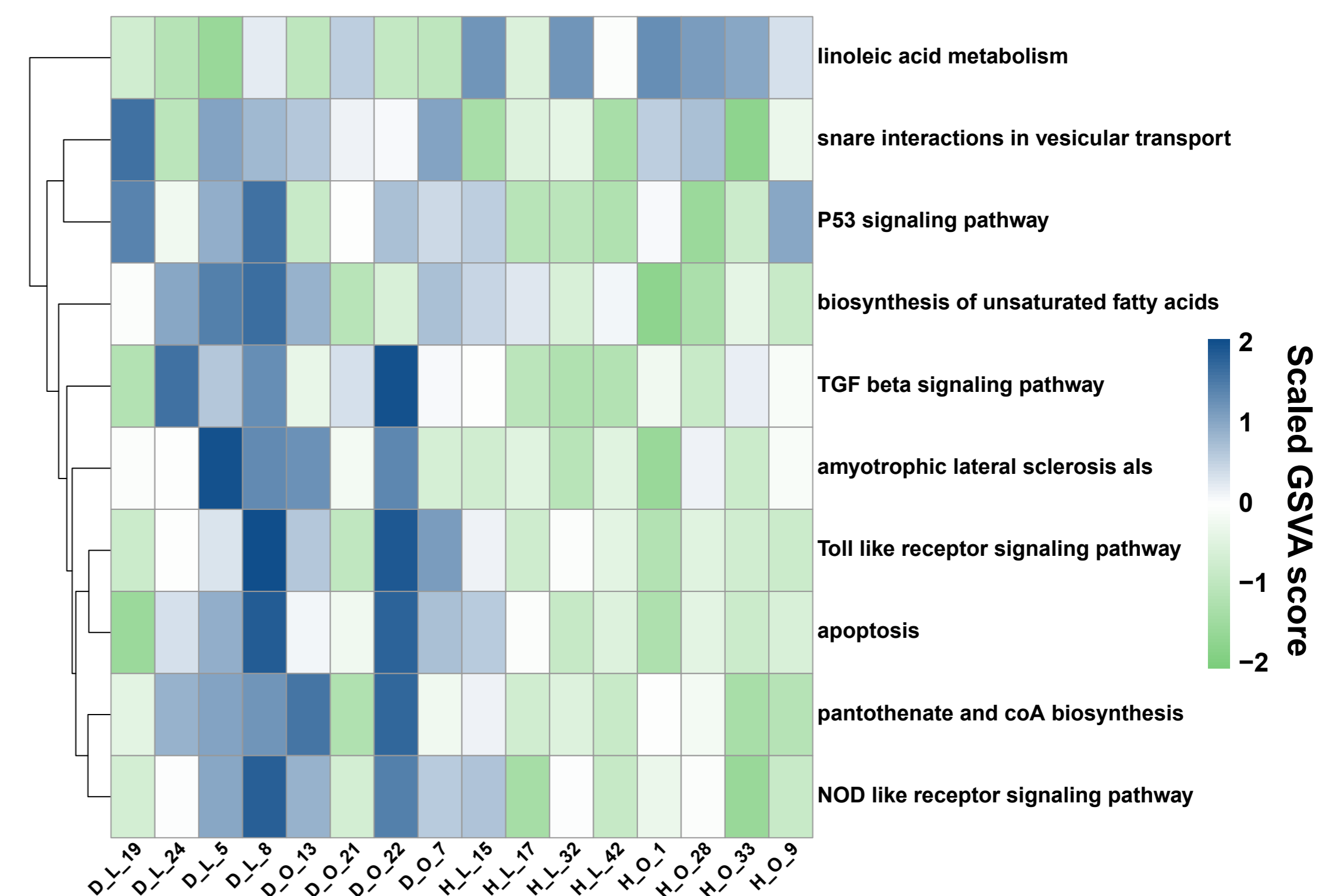

Supplement: Supplementary Materials — Supplementary figures depict the differentially expressed gene sets (Figure S1) and the PPI network (Figure S2). [file 6793346.f1.zip › Figure S1.pdf]

A

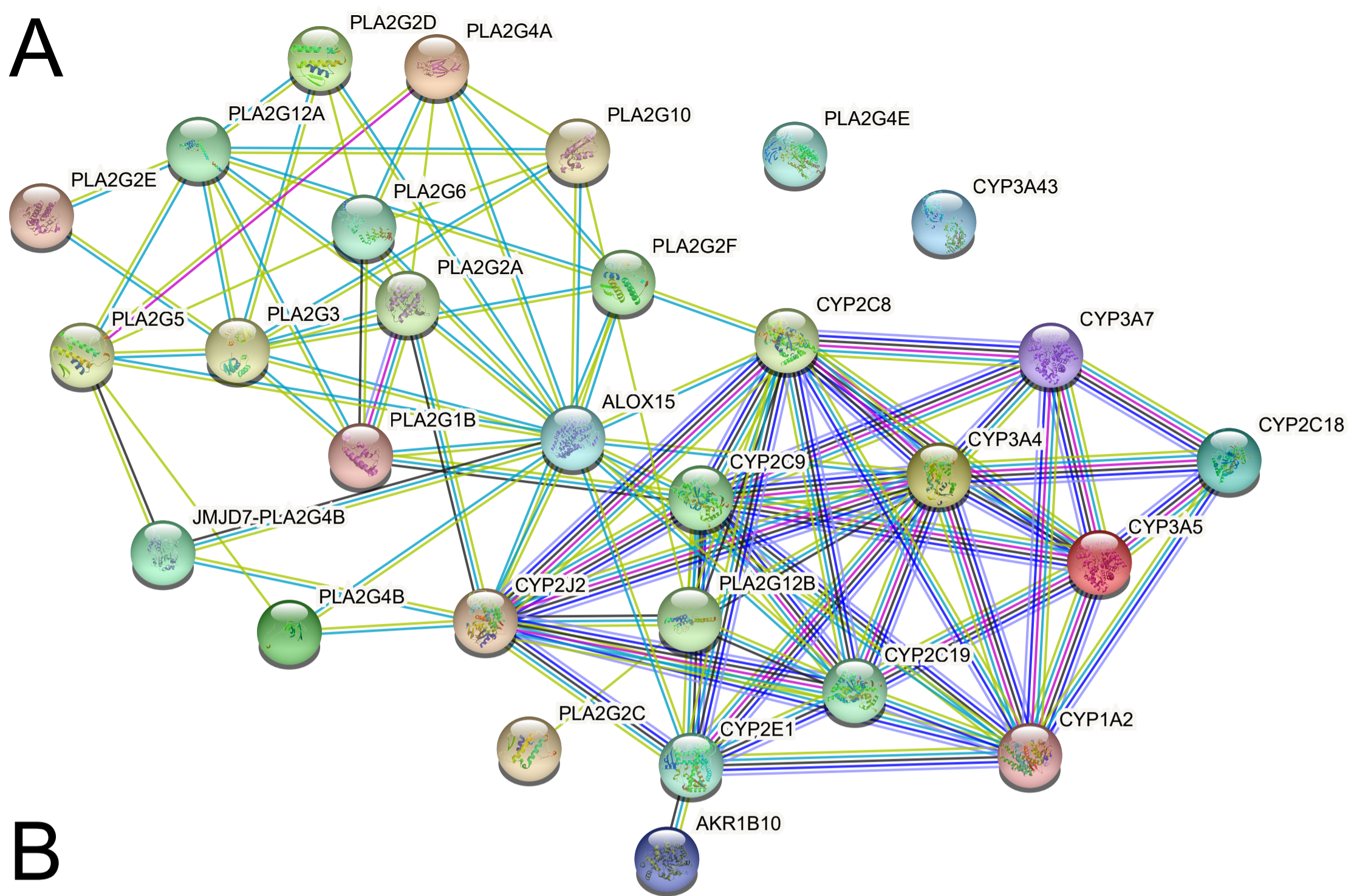

B

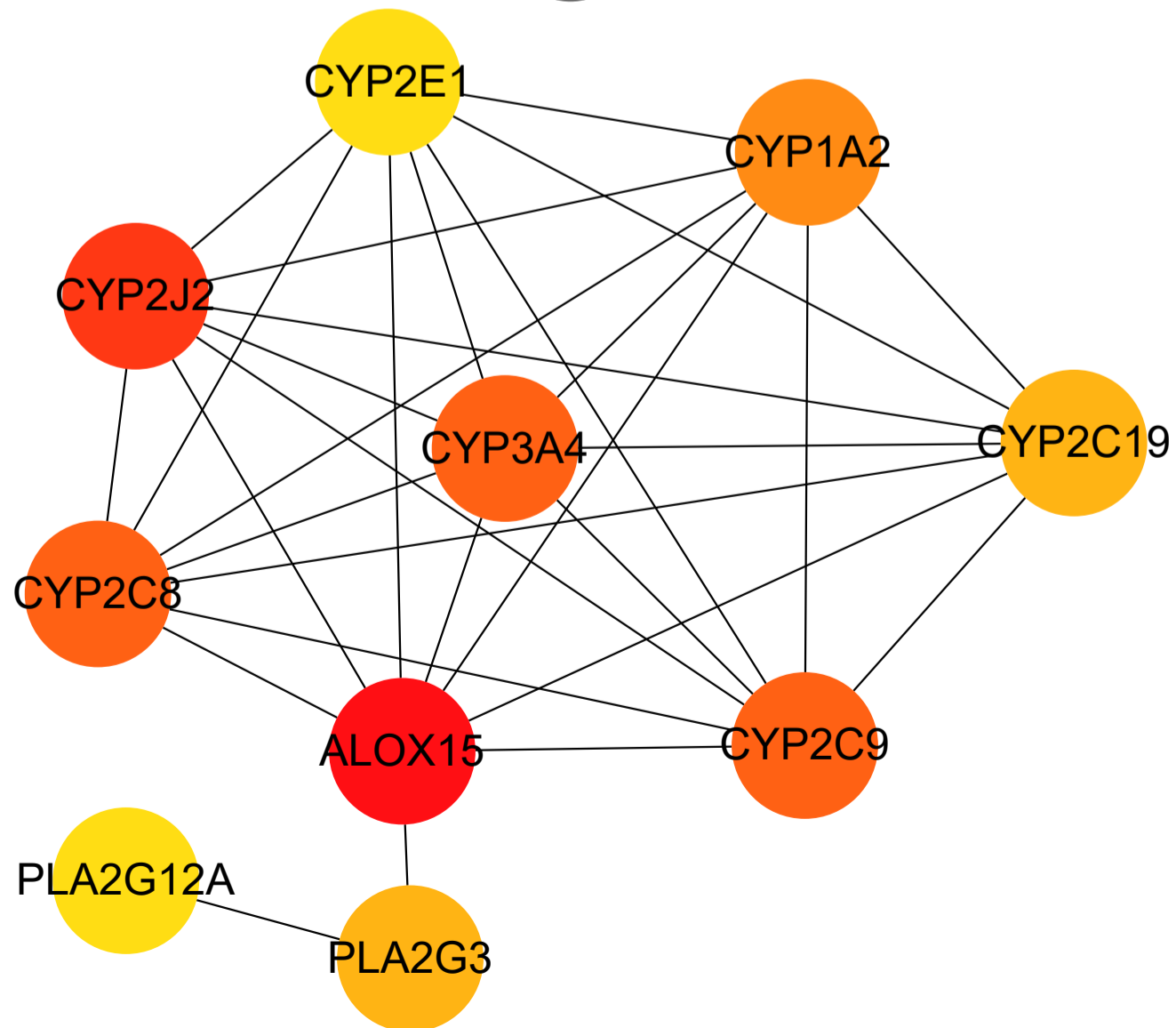

Supplement: Supplementary Materials — Supplementary figures depict the differentially expressed gene sets (Figure S1) and the PPI network (Figure S2). [file 6793346.f1.zip › Figure S2.pdf]
